# Foliar Application of Melatonin Improves the Salt Tolerance, Ion and Redox Homeostasis and Seed Oil Fatty Acid Profile in *Camelina sativa*

**DOI:** 10.3390/plants11223113

**Published:** 2022-11-15

**Authors:** Mohammad Reza Forozan Bakyani, Mozhgan Alinia, Seyed Abdolreza Kazemeini, Javier Abadía, Ali Dadkhodaie

**Affiliations:** 1 Department of Plant Production and Genetics, Shiraz University, Shiraz 71441-13131, Iran; 2 Department of Plant Biology, Aula Dei Experimental Station (CSIC), Av. Montañana 1005, 50059 Zaragoza, Spain

**Keywords:** antioxidant enzyme activity, gas exchange, ion-selective transport, unsaturated fatty acids

## Abstract

Salinity affects the yield and quality of oilseed crops. The effects of a single foliar application of solutions with different concentrations (0, 30, 60 or 90 µM) of melatonin (MEL) to camelina (*Camelina sativa*) plants grown in soil in a greenhouse and irrigated at four salinity levels (0.5, 4, 8 and 16 dS m^−1^) were assessed. Increasing salinity decreased leaf chlorophyll and photosynthetic rates, decreased K concentrations and increased Na concentrations in roots and shoots, and increased oxidative marker levels and the activity of protective antioxidant enzymes in leaves. Under severe salinity stress, the MEL90 treatment resulted in increases in chlorophyll, gas exchange attributes, leaf antioxidant enzyme activities, and decreases in leaf oxidative markers and Na. Salinity decreased seed yield, with no seeds being produced at salinities above 8 dS m^−1^. The MEL90 treatment resulted in increases in seed yield and poly- and mono-unsaturated fatty acid contents and decreases in saturated fatty acid contents. The MEL90 treatment was more effective in alleviating salinity effects than those including lower MEL concentrations. The highest concentrations of K and K/Na ratios were observed with the MEL90 treatment under non-stressed conditions. Data suggest that MEL foliar applications could increase salinity stress tolerance in camelina.

## 1. Introduction

Camelina (*Camelina sativa* (L.) Crantz) is an industrial oilseed species (Brassicaceae) that is mainly grown in marginal lands unsuitable for food crop production [1,2]. Camelina is widely used in the bio-lubricant, biofuel, and animal feed sectors because it contains considerable amounts of protein (ca. 30%), fiber (ca. 12%), and especially oil (35–45%) [3]. The oil obtained from camelina seeds mainly contains unsaturated fatty acids (ca. 90%), and low levels of erucic acid [4,5]. Camelina is moderately sensitive to salinity stress at critical growth stages [6].

Soil and/or irrigation water salinity decrease crop yields worldwide in arid and semi-arid regions. Salinity stress can elicit a myriad of molecular, biochemical and physiological alterations in plants, with its severity and duration determining the extent of plant damage. Salinity usually leads to large deleterious effects on plant development and growth, causing ion imbalance and osmotic stress, and eliciting oxidative stress via changes in molecular and biochemical pathways [7,8]. A possible way to alleviate the harmful effects of salinity would be to design and use management strategies that can increase the plant potential to maintain tolerance and productivity under this stress.

Plant breeding and genetic engineering are good approaches to increase the tolerance of crops to different abiotic stresses, but they are costly and are not likely to be effective in the short term [8,9]. Alternatively, other more rapid and less expensive biological techniques can be employed to improve agricultural crop yields under severe environmental conditions [10]. For instance, using plant growth regulators and other substances can improve tolerance to abiotic stress at different plant developmental stages [11]. These substances act through a range of mechanisms, including the enhancement of nutrition efficiency and crop quality traits.

Melatonin (MEL) is an indole-type compound, structurally related to indole-3-acetic acid and serotonin. Exogenous applications of MEL have been shown to increase the tolerance of plants to stress conditions, including salinity [10,12,13]. This has been reported for strawberry [14], faba bean [15], common bean [16], and rapeseed [17,18]. In these studies, it was shown that the application of MEL elicited increases in the activity of enzymatic antioxidants and decreases in the levels of reactive oxygen species (ROS), thus mitigating oxidative stress, improving gas exchange parameters and photosynthesis and maintaining ion homeostasis. For instance, the application of MEL in rapeseed grown under salinity conditions improved H_2_O_2_ scavenging by enhancing the leaf levels of antioxidant enzymes and alleviated osmotic stress via an increase in osmoregulatory substances [19]. The oil quality of rapeseed was reduced under salinity stress due to increases in the content of glucosinolates and saturated fatty acids (palmitic and arachidic acids) and decreases in unsaturated fatty acids (linolenic and oleic acids, but not erucic acid), and the application of MEL was effective in alleviating these effects [20]. Also in rapeseed, MEL was shown to play an essential role in reducing salinity-induced malondialdehyde and electrolyte leakage, hence improving redox modulation and ion homeostasis [21].

In this study, we tested the hypothesis that a single foliar application of MEL can improve the salt tolerance, the ion and redox homeostasis and the seed fatty acid profile in camelina. There is currently no information regarding the effects of foliar MEL applications in camelina plants grown under salinity conditions. Therefore, the objectives were to assess the effects of a single MEL foliar application on the seed yield of camelina irrigated with water of different salinity levels; to characterize the changes in gas exchange parameters, antioxidant enzyme activities, ion homeostasis and seed fatty acid profiles in response to salinity and MEL; and to obtain information on the mechanisms through which MEL can alleviate salinity stress in camelina.

## 2. Results

### 2.1. Leaf Photosynthetic Pigments

Increases in salinity led to progressive decreases in the leaf photosynthetic pigment concentrations when compared with the values observed in control conditions (0.5 dS m^−1^), and the foliar application of 90 µM MEL made the decreases less intense (Table 1). The highest total Chl and carotenoid concentrations (2.95 and 2.85 mg g^−1^ FW) were observed with the MEL90 treatment in control conditions (0.5 dS m^−1^), and the minimum ones (1.73 and 0.42 mg g^−1^ FW) were found in the 16 dS m^−1^ salinity level in the absence of MEL. When salinity was 4, 12 and 16 dS m^−1^, the foliar MEL90 treatment improved the total Chl by 1.1-, 1.1-, and 1.2-fold, respectively, when compared to the zero MEL treatments. The carotenoid concentrations increased in all salinity levels (by 1.6-, 2.0-, 1.9-, 2.3- and 2.1-fold in the cases of the 0.5, 4, 8, 12 and 16 dS m^−1^, respectively), when compared to the zero MEL treatments. The increases in Chl and carotenoid concentrations with MEL60 and MEL30 under salinity stress were less intense than those obtained with MEL90. The analysis of variance showed significant interactions between MEL application rates and salinity levels in the cases of total Chl and carotenoids.

### 2.2. Leaf Gas Exchange Parameters

Increases in salinity led to progressive decreases in P_n_, Tr and g_s_, and increases in C_i_ when compared with the values observed in the control condition, and the foliar application of MEL at all concentrations (30, 60 and 90 µM) attenuated these changes (Table 1). In the 0.5, 4, 8, 12 and 16 dS m^−1^ salinity levels, MEL90 increased P_n_ by 3.6-, 4.3-, 4.8-, 6.3- and 8.5-fold, Tr by 1.5-, 1.7-, 1.8-, 1.8- and 1.9-fold, and g_s_ by 1.5-, 1.8-, 1.8-, 2.2- and 2.6-fold, when compared to the values observed in the absence of MEL. On the other hand, the MEL90 treatment decreased C_i_ by 25, 24, 22, 22 and 20%, respectively, in the 0.5, 4, 8, 12 and 16 dS m^−1^ salinity treatments. Under salinity stress conditions, the 90 µM treatment was more effective than the 30 and 60 µM ones when compared to the zero MEL application. The analysis of variance showed significant interactions between MEL application rates and salinity levels for all gas-exchange parameters.

### 2.3. Antioxidant Enzyme Activities in Leaf Extracts

**Figure 1:**
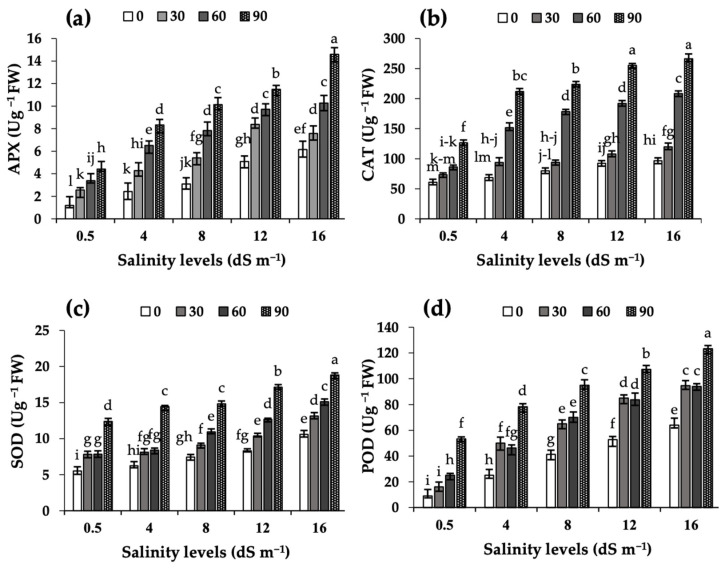
Effects of MEL foliar applications (0, 30, 60 and 90 µM) on APX (**a**), CAT (**b**), SOD (**c**), and POD (**d**) enzyme activities in leaf extracts of camelina grown under different levels of salinity (0.5, 4, 8, 12 and 16 dS m^−1^). APX: ascorbate peroxidase; CAT: catalase; SOD: superoxide dismutase, and POD: peroxidase. Data shown are means of three replicates per treatment ± SD. Different letters above the columns indicate statistically significant differences at *p* < 0.05.

Increases in salinity led to progressive increases in the APX, CAT, SOD, and POD activities in leaf extracts of camelina when compared with the values observed in the control conditions, and these activities further increased progressively with MEL application rates (Figure 1). In the absence of MEL treatments, the 16 dS m^−1^ salinity treatment resulted in 5.2-, 1.6-, 1.9- and 7.1-fold increases in the activity of APX, CAT, SOD and POD, respectively, when compared to values in the control conditions. The highest activity values (all expressed in U g^−1^ FW) occurred in the MEL90 treatment under 16 dS m^−1^ salinity (14.6 for APX, 266.3 for CAT, 18.8 for SOD and 123.3 for POD).

### 2.4. Malonyldialdehyde (MDA) and Hydrogen Peroxide (H_2_O_2_) in Leaves

**Figure 2:**
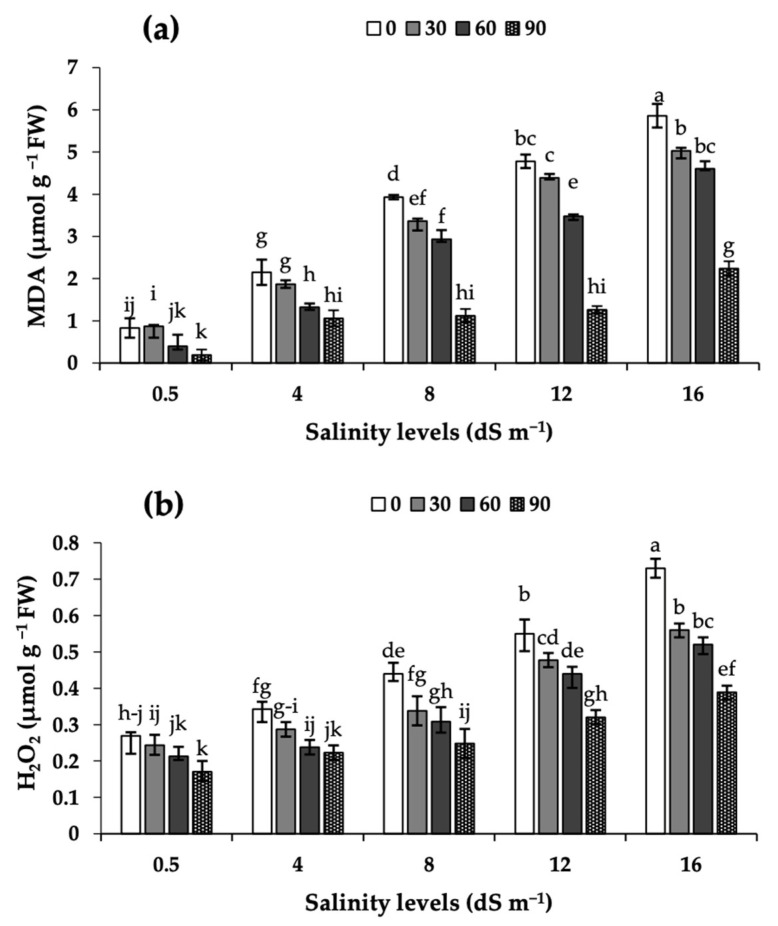
Effects of MEL foliar applications (0, 30, 60 and 90 µM) on MDA (**a**), and H_2_O_2_ levels (**b**) in leaves of camelina grown under different levels of salinity (0.5, 4, 8, 12, and 16 dS m^−1^). MDA: malonyldialdehyde, and H_2_O_2_: hydrogen peroxide. Data shown are means of three replicates per treatment ± SD. Different letters above the columns indicate statistically significant differences at *p* < 0.05.

The damage to the cell membrane and lipid peroxidation in camelina leaves were investigated in terms of the changes in the leaf H_2_O_2_ and MDA levels (Figure 2). Increases in salinity led to progressive increases in the MDA and H_2_O_2_ levels, and MEL foliar applications led to decreases in these parameters, even under severe salinity stress. The decreases in the H_2_O_2_ level were largest (47%) when MEL90 was applied in the highest salinity level (16 dS m^−1^).

### 2.5. Ion Concentrations, Translocation and Selective Transport (ST)

The effect of salinity on the K and Na concentrations in roots and shoots, and the translocation and selective transport in camelina plants are shown in Table 2. Increases in salinity led to progressively large increases in Na levels in shoots and roots when compared with the values observed in control conditions, whereas the opposite occurred for K and the K/Na ratio. The shoot and root concentrations of K and Na were markedly affected by the MEL foliar applications, and in salinity-stressed plants, a single application of MEL90 led to a marked increase in K concentrations, and an even greater decrease in the concentrations of Na in both the aerial and underground parts. The highest concentration of Na was found in the plants grown in the 16 dS m*^−^*^1^ salinity level in the absence of MEL (8.9 and 10.3 mg g*^−^*^1^ DW in shoots and roots, respectively), and the lowest ones were observed in the MEL90 and the control treatments (0.5 and 1.9 mg g*^−^*^1^ DW in shoots and roots, respectively). The application of MEL90 markedly increased selective ion transport due to the increases in K concentrations. Additionally, the application of MEL90 led to increases in the translocation of K, resulting in the accumulation of this element in shoots. The accumulation of K in plants treated with MEL90 was much higher than in those with lower MEL concentrations. The analysis of variance showed significant interactions between MEL application rates and salinity levels for all nutrient parameters.

### 2.6. Seed Yield

**Figure 3:**
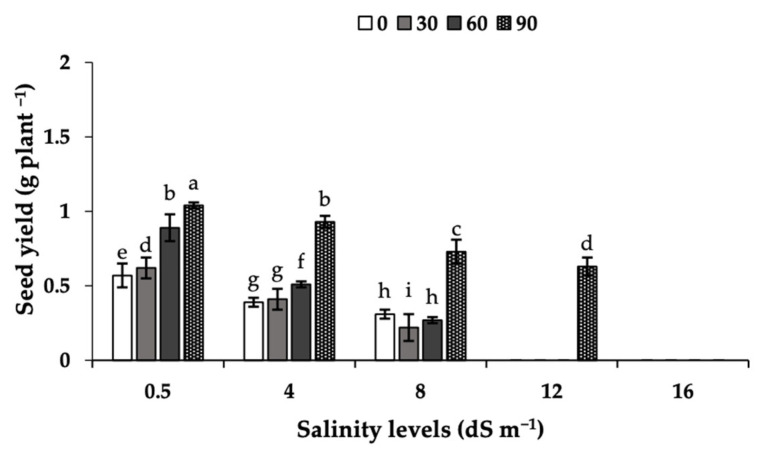
Effects of MEL applications (0, 30, 60, and 90 µM) on seed yield (g plant^−1^) in camelina plants grown under different levels of salinity (0.5, 4, 8, 12, and 16 dS m^−1^). Data shown are means of three replicates per treatment ± SD. Different letters above the columns indicate statistically significant differences at *p* < 0.05.

In plants grown at 4–8 dS m^−1^, there was a marked decrease in seed yield when compared to those in the control, whereas in plants grown at 12 and 16 dS m^−1^, no seeds were produced (Figure 3). The foliar application of MEL had a marked positive effect on seed yield, and seeds were produced with MEL90 in the 12 dS m^−1^ salinity treatment (0.63 g plant^−1^). In all cases, the seed yield was higher with MEL90 than with the other MEL treatments. The highest seed yield value (1.04 g plant^−1^) was observed in the MEL90-treated plants under control conditions.

### 2.7. Seed Fatty Acid Profile

The fatty acid profile of camelina seeds changed with the salinity and also by the MEL foliar applications (Table 3). The relative contents of palmitic and stearic acids (C16:0 and C18:0, respectively), the main saturated fatty acids (SFA), increased progressively with salinity, and were particularly high at 8 dS m^−1^, whereas those of mono- and poly-unsaturated fatty acids (MUFA and PUFA, respectively) levels decreased progressively with increasing salinity. When compared to the control, decreases of 76, 78, 100, 8, 67, 36 and 72% were observed for oleic (C18:1), eicosenoic (C20:1), erucic (C22:1), linoleic (C18:2), linolenic (C18:3), eicosadienoic (C20:2) and eicosatrienoic (C20:3) acids in plants grown in the 8 dS m^−1^ salinity treatment in the absence of MEL. At the 4 and 8 dS m^−1^ salinity levels, a single foliar application of MEL90 increased the relative contents of MUFA and PUFA when compared to those in the absence of MEL. At the 8 dS m^−1^ salinity level, the relative contents of oleic, eicosenoic, erucic, linoleic, linolenic, eicosadienoic and eicosatrienoic acids increased from 3.90, 0.52, 0.00, 21.59, 15.61, 0.23 and 0.05% in the absence of MEL to 14.56, 4.77, 0.20, 23.72, 42.17, 0.82 and 0.42% in the MEL90 treatment.

## 3. Discussion

Salinity is a major environmental constraint that negatively impacts crop production. Salinity stress leads to redox imbalance between the generation of ROS and antioxidants, resulting in oxidative damage as well as ionic imbalance and ion toxicity. Data show that irrigating camelina plants with saline water decreased significantly the camelina seed yield, with no seeds being obtained at salinities of 12 and 16 dS m^−^^1^. In a previous study, it was shown that salinity stress led to a significant reduction in camelina yield, reducing the percentage of emergence and growth attributes [22]. In many other crop plants, previous studies also described the deleterious effects of salinity on yield, with the extent of losses usually depending on the salinity level and the crop growth stage [15,23]. The camelina seed oil fatty acid composition was also markedly changed by irrigation water salinity, with relative increases and decreases in saturated (SFA) and unsaturated fatty acids (MUFA and PUFA), respectively. Previous studies have shown that the unsaturated fatty acids content decrease with stress in some oil crops, including coriander [24] and camelina [25]. In rapeseed, salinity stress at the seed development stage was found to decrease photosynthetic assimilates and carbon partitioning to seeds and increase the activities of enzymes involved in fatty acid oxidation [20].

The salinity-induced decline in camelina yield was associated with the occurrence of ROS in leaves, as judged by the increases in H_2_O_2_ contents and lipid peroxidation as well as the increases in the activity of enzymatic antioxidants (APX, CAT, SOD, and POD), and with decreases in leaf photosynthetic pigment concentrations, photosynthesis and transpiration rates and stomatal opening. Previous studies in other crops showed that salinity stress leads to increases in H_2_O_2_ and MDA [7,12], and that the accumulation of ROS generally caused increases in the activity of APX, CAT, SOD, and POD [26,27]. In previous studies with other crop species, a reduction in photosynthetic pigments was often found [16,21,28]. These decreases were associated with increases in the expression of the gene coding for chlorophyllase, which is involved in chlorophyll degradation, in faba bean [15], common bean [23] and camelina [29].

Melatonin is a natural bio-stimulating substance, involved in regulating plant growth, development and yield [10]. In this study, it was found that a single MEL application in the control treatment (0.5 dS m^−^^1^) already caused significant increases in seed yield, increases in the activities of antioxidant enzymes, as well as decreases in oxidative markers and increases in net photosynthesis. These effects were more marked with 90 µM MEL than with lower MEL concentrations. These data are in line with those recently observed in strawberry [14]. In previous studies with common bean [16] and rapeseed [17], MEL treatments also led to improvements in physiological and biochemical traits and seed yield. The reason behind the positive effects of MEL in field conditions in the absence of salinity is still unknown, but it could be speculated that they may be related to the beneficial effect of this compound on the plant redox status.

Enzymatic antioxidants act as ROS scavengers and decrease oxidative damage [7]. Data show that a single foliar MEL application to camelina plants improved tolerance to salinity, and that this improvement was accompanied by a relief in oxidative stress, as judged from the decrease in the levels of oxidative markers (H_2_O_2_ and MDA) and the activities of redox-protecting enzymes (APX, CAT, SOD, and POD). These effects depend on the MEL dose and were largest with the MEL90 treatment. CAT and SOD are key enzymes involved in cell-defense processes that detoxify oxygen radicals and mitigate the effects of salinity [30]. The MDA level is usually considered as a good proxy for the degree of lipid peroxidation in the cell membrane [31]. Melatonin treatments are thought to play a multifunctional role in improving stress tolerance, and seed yield is considered an overall indicator of metabolism functioning in plants. The application of MEL in plants grown under salinity often leads to increases in the efficiency of the mitochondrial electron transport chain and to decreases in the generation of free radicals [9]. Data shown here are in line with those reported in recent studies with MEL application to plants cultivated under stress conditions. For instance, MEL application has been shown to lead to significant increases in the drought tolerance of rapeseed [18], and to alleviate the reductions caused by salinity in growth parameters and yield of common bean [16]. Additionally, in rapeseed grown under salinity stress, the application of MEL enhanced significantly the activity of enzymatic antioxidants, such as APX, CAT, SOD, and POD [20]. Increases in antioxidant enzymes and decreases in the contents of H_2_O_2_ and MDA with MEL application in crops grown under abiotic stresses have also been consistently reported [13,32]. The beneficial effects of MEL in plant redox status have been found in maize [33], faba bean [15], tomato [34] and rapeseed [17].

Chlorophylls and carotenoids are important pigments that carry out the absorption of light for plant photosynthesis [16]. Leaf photosynthetic pigment (Chl and carotenoid) concentrations and photosynthetic parameters were also improved by MEL foliar applications. The P_n_, Tr, and g_s_ decreases caused by salinity were alleviated by foliar applications of MEL90. In previous studies, MEL applications were shown in many crops to improve light energy absorption and photosystem II electron transport, increase Chl concentrations, and enhance carbon fixation by improving stomatal function and increasing Rubisco enzyme activity [16,20,32]. Increases in leaf photosynthetic pigments and photosynthetic rates with MEL application in crops grown under salinity stress have been consistently reported in maize [35], tomato [34], rapeseed [20] and common bean [15,16]. In plants grown under salinity stress, the application of MEL improved significantly the stomatal function and decreased significantly the expression of the gene coding for chlorophyllase [15,35].

Ion homeostasis is an important mechanism that can alleviate the deleterious effects of salinity stress by regulating ion uptake and transport [13]. Data show that foliar MEL90 application to camelina plants affected by salinity improved tolerance to this stress, and this improvement was accompanied by increases in K concentration, K/Na ratio, and ion selective transport. The application of MEL caused changes in ion homeostasis, leading to an alleviation of the effects of salinity by facilitating the transport of ions from the cytoplasm to the vacuole and/or their compartmentalization in specific tissues. In previous studies, increases in the K concentration, K/Na ratio, and ion selective transport and regulation of *NHX* expression were consistently observed in several crops with MEL application under abiotic stresses [16,33,36,37].

The aim of camelina growers is to improve not only the seed yield, but also the quality of the oil extracted from seeds. In the case of camelina, the seed oil quality mainly depends on the fatty acid composition, particularly regarding unsaturated fatty acids (oleic, eicosenoic, erucic, linoleic, linolenic, eicosadienoic and eicosatrienoic acids), and this can be highly influenced by the environment [25]. The oil quality increases when higher relative contents of MUFA and PUFA and lower relative contents of SFA occur. Data show that a single foliar application of MEL90 to camelina plants irrigated with saline water increased MUFA and PUFA relative contents and reduced SFA relative contents in seeds. In a previous study with rapeseed, MEL foliar applications were shown to result in the activation of fatty acid biosynthesis enzymes [20].

## 4. Materials and Methods

### 4.1. Plant Material

The experiment was conducted at the School of Agriculture, Shiraz University (1810 m above mean sea level), in March 2021. The origin of camelina seeds (*Camelina sativa* L. Crantz, cultivar Soheil) was the Seed and Plant Improvement Institute (Karaj, Iran). Camelina was cultivated in a greenhouse with a temperature of 29 ± 2 °C, 80% RH and natural photoperiod.

### 4.2. Experimental Design

The study was performed as a factorial experiment with two factors in a completely randomized design. Four MEL concentrations and five levels of salinity, with three replicates, were used. Melatonin (*N*-acetyl-5-methoxytryptamine; Sigma Aldrich Chemie, Steinheim, Germany) was dissolved in a small volume of ethanol, and solutions with final MEL concentrations of 0, 30, 60, and 90 µM were prepared with distilled water. Seeds were sowed (10 per pot) at 1 cm depth in plastic pots (20 × 18 cm, inner diameter × depth) with 4.5 kg of soil. The soil used was collected from the field (depth of 0–30 cm) at the Shiraz University Campus, and had a loamy texture (29.6% sand, 44.0% silt and 26.4% clay), a pH of 7.20 and an electrical conductivity (EC) of 0.64 dS m^−1^. Other soil characteristics were 11.6 cmol kg^−1^ cation exchange capacity, 1.2% organic matter, 59% CaCO_3_, 0.12% total N, 5 mg kg^−1^ extractable P and 198 mg kg^−1^ extractable K. In order to provide nutrients at sufficient levels, all pots received 25 mg P kg^−1^ soil as Ca(H_2_PO_4_)_2_.H_2_O, 45 mg N kg^−1^ soil as NH_4_NO_3_, and 6 mg Fe kg^−1^ soil as Fe(III)-EDDHA. Each pot was irrigated with tap water (650 mL per pot) from the city supply (EC 0.5 dS m^−1^) on a weekly basis until the beginning of the salinity stress treatments. After emergence, only five seedlings were left in each pot. At 33 days after sowing (DAS) (BBCH scale code-13), plants had six fully expanded leaves, and were submitted to five irrigation water salinity treatments: 0.5 (control), 4, 8, 12, and 16 dS m^−1^ (using 0, 2.5, 5.1, 7.7, and 10.2 g L^−1^ NaCl in tap water, respectively). Plants were irrigated weekly with the saline solutions, and treatments were maintained for 60 days. Two days after the start of the salinity stress treatments, the adaxial leaf surfaces of all plants were sprayed with the MEL solutions until they were fully wet (21 mL plant^−1^; this corresponds to approximately 0.15, 0.29 and 0.44 mg MEL per plant in the cases of the 30, 60, and 90 µM MEL treatments). Camelina has been shown to have stomata in the abaxial and adaxial leaf surfaces [38]. At sampling time, two plants per pot were sampled to measure the biomass and biochemical characteristics, and the remaining three were kept until seed maturity (100 DAS) to assess yield-related traits.

### 4.3. Leaf Chlorophyll and Carotenoid Analysis

Leaf samples were taken at 67 DAS (BBCH scale code-60) from 10:00 to 12:00 AM. Leaf tissue was frozen in liquid N_2_ and stored at −80 °C. The leaf concentrations of chlorophylls (Chl) and carotenoids were measured spectrophotometrically [39,40]. Leaf tissues (0.1 g fresh weight -FW-) were homogenized in 10 mL of 80% acetone in the dark until the residue was colorless. The homogenate was spun at 500 rpm for 5 min at room temperature (RT), and A_646_, A_663_, and A_470_ were measured (7315 UV/VIS, Jenway, Staffordshire, UK). Then, pigment concentrations were determined as follows:

Chl a = 12.21 A_663_ − 2.81 A_646_

Chl b = 20.13 A_646_ − 5.03 A_663_

Total Chl = Chl a + Chl b

Carotenoids = (1000 A_470_ − 3.27 Chl a − 104 Chl b)/198

### 4.4. Photosynthetic Parameter Determination

The sub-stomatal CO_2_ concentration (C_i_), transpiration rate (Tr), stomatal conductance (g_s_), and net photosynthesis rate (P_n_) were measured in fully expanded terminal leaflets of each plant (LCi, ADC Bioscientific Ltd., Hoddesdon, UK). All measurements were made at 72 DAS (BBCH scale code-63) from 9.00 to 11.00 AM.

### 4.5. Antioxidant Analysis

Leaf tissue was sampled at 67 DAS to determine the activities of antioxidant enzymes. Leaf extracts were made using 0.5 g of leaf tissue in 5 mL of ice-cold phosphate buffer (pH 7.6), and all determinations were made spectrophotometrically (7315 UV/VIS, Jenway) using phosphate buffers. The peroxidase (POD) activity was determined from the changes in A_470_ using an assay mixture containing 50 mM buffer (pH 7.0), 16 mM guaiacol and 0.2 mL of sample, after adding 10 mM H_2_O_2_ [41]. The superoxide dismutase (SOD) activity in leaf extracts was measured in a solution containing 50 mM buffer (pH 7.6), 750 mM NBT, 4 μM riboflavin, 13 mM methionine, 0.1 mM EDTA and 0.2 mL of extract [42]. Cuvettes containing the assay solution were illuminated with fluorescent lamps for 15 min, and the photochemical reduction of NBT was followed from the changes in A_560_. The catalase (CAT) activity was measured at A_240_ using an assay solution containing 50 mM buffer (pH 7.0) and 12.5 mM H_2_O_2_, mixed with 0.2 mL of sample [43]. The ascorbate peroxidase (APX) activity was determined at A_290_ using an assay solution containing 50 mM buffer (pH 7.0), 0.1 mM EDTA, 0.5 mM ascorbate (AsA), 1.0 mM H_2_O_2_ and 0.2 mL of sample [44].

### 4.6. Malondialdehyde (MDA) and H_2_O_2_ Content Determination

The lipid peroxidation in leaves was determined from the MDA contents [45]. Fresh leaf tissue (0.2 g) was homogenized, and 5 mL of 1% trichloroacetic acid (TCA) was added. The homogenized sample was spun at 8000 rpm for 10 min. Then, 1 mL of supernatant was mixed with 4 mL of 20% TCA and 0.5% thiobarbituric acid. Samples were kept at 95 °C for 30 min, immediately placed in iced water and spun at 8000 rpm for 5 min. Measurements were taken at A_532_ and A_600_. The content of MDA was measured, using an extinction coefficient of 155 mM^−1^ cm^−1^, as

$$\mathrm{MDA}\;\left({\mu \mathrm{mol}\;g}^{-1}\;\mathrm{FW}\right)=\left[A_{532}-\frac{A_{600}}{155}\right]\times 1000$$

For hydrogen peroxide (H_2_O_2_) determination, fresh leaf tissue (0.5 g) was homogenized in an ice bath with 5 mL 0.1% TCA [46]. The homogenate was spun at 12,000 rpm for 15 min, and 0.5 mL of the supernatant was added to 0.5 mL of 10 mM K buffer (pH 7.0) and 1 mL of 1 M KI. Measurements were taken at A_390_ using a spectrophotometer.

### 4.7. Ion Concentrations, Translocation, and Selective Transport

Oven-dried shoot and root samples (0.5 g) were milled using a grinder, ashed in an electric oven (at 450 °C for 4 h) and then digested with 1 N HCl. The tissue concentrations of K and Na were determined by flame photometry [47]. Potassium and Na translocation from roots to shoots, and selective transport (ST) were determined using the following equations [48,49]:

$$\mathrm{Ion}\;\mathrm{translocation}\;\mathrm{factor}=\frac{\mathrm{Ion}\;\mathrm{concentration}\;\mathrm{in}\;\mathrm{shoots}}{\mathrm{Ion}\;\mathrm{concentration}\;\mathrm{in}\;\mathrm{roots}}$$

$$\mathrm{ST}=\frac{\mathrm{Na}/K\;\mathrm{in}\;\mathrm{roots}}{\mathrm{Na}/K\;\mathrm{in}\;\mathrm{shoots}}$$

### 4.8. Seed Yield and Fatty Acid Profile Analysis

At the full-ripening stage (100 DAS), the remaining three plants per pot plants were harvested, and the seed yield per plant was measured. The fatty acid profile of seeds was determined as in Golmakani et al. [50]. Seeds (500 mg) were ground in 10 mL of methanol:acetyl chloride (95:5, *v*/*v*). The homogenate was placed at 85 °C for 1 h, cooled to RT, 5 mL of distilled water was added, and the mixture vortexed for 5 min. Then, 2 mL of hexane with 0.01% tertiary butylhydroquinone was added, and the mixture shaken for 5 min to prevent oxidation. The sample was spun at 4000 rpm for 5 min at 25 °C. The hexane layer upper phase contained a mixture of fatty acid methyl esters, which were then quantified (SP-3420A gas chromatograph, Beijing Beifen-Riuli Analytical Instrument Group, Beijing, China), using a split ratio of 1:10 and a silica capillary-column (BPX70, 120 m × 0.25 mm × 0.25 µm).

### 4.9. Statistical Analysis

Statistical analysis was carried out using SAS v. 9.1 software (SAS Institute 2003), and means for each trait were compared using the LSD test (*p* < 0.05).

## 5. Conclusions

Data confirm the hypothesis that a single foliar application of MEL improved salinity tolerance in camelina, increasing the activities of antioxidant enzymes, the leaf photosynthetic pigment levels and the photosynthesis rates, decreasing the MDA and H_2_O_2_ content, and increasing the leaf K concentrations and the K/Na ratio, as well as the translocation rate and the ST. Results also confirm the hypothesis that a single MEL application can improve the seed yield and change the fatty acid profile in salinity stressed plants, increasing the relative proportion of unsaturated fatty acids, therefore resulting in a better oil quality. The effects were largest with the highest MEL dose (MEL90). These findings support that the foliar application of MEL can be envisaged as a relevant strategy to improve the salt tolerance as well as the yield and quality of seed oil in camelina plants irrigated with saline water. Considering the plant density used in a similar *Brassicaceae* species (approximately 100 plants per m^2^; pennycress, *Thlaspi arvense*; [51]), the MEL doses used in this study (0.15, 0.29 and 0.44 mg MEL plant^−1^) would be equivalent to approximately 146, 293 and 439 g MEL ha^−1^. Therefore, the possibility of using MEL in field crops will largely depend on the commercial price of this compound. Further research should focus on the possibility of using wholesale MEL (much cheaper than pharmaceutical grade), as well as its possible use in seed priming, which would likely involve lesser amounts, instead of using foliar applications. On the other hand, the possible effects of MEL on root characteristics were not assessed in this study and deserve further investigation.

## Figures and Tables

**Table 1:** Effects of MEL foliar applications (0, 30, 60 and 90 µM) on the leaf photosynthetic pigment concentrations and gas exchange attributes of camelina grown under different levels of salinity (0.5, 4, 8, 12 and 16 dS m^−1^).

Salinity Level (dS m^−1^)	MEL (µM)	Total Chl (mg g^−1^ FW)	Carotenoids (mg g^−1^ FW)	P_n_ (µmol CO_2_ m^−2^ s^−1^)	Tr (mmol H_2_O m^−2^ s^−1^)	g_s_ (mol H_2_O m^−2^ s^−1^)	C_i_ (µmol CO_2_ mol ^−1^)
0.5	0	2.88 ^ab^	1.76 ^c^	3.65 ^i^	3.38 ^f–h^	0.21 ^d–g^	353.33 ^e^
	30	2.89 ^ab^	2.34 ^b^	8.56 ^de^	4.04 ^c–e^	0.27 ^a–c^	326.00 ^f^
	60	2.89 ^ab^	2.40 ^b^	9.28 ^d^	5.06 ^ab^	0.30 ^ab^	296.50 ^gh^
	90	2.95 ^a^	2.85 ^a^	13.25 ^a^	5.22 ^a^	0.32 ^a^	264.50 ^i^
4	0	2.51^ef^	0.91 ^f–h^	3.00 ^ij^	2.69 ^i–k^	0.14 ^i–k^	376.33 ^d^
	30	2.63 ^de^	0.96 ^e–h^	7.91 ^ef^	3.35 ^gh^	0.20 ^d–h^	349.00 ^e^
	60	2.65 ^cd^	1.36 ^d^	8.63 ^de^	4.37 ^cd^	0.23 ^c–e^	319.50 ^f^
	90	2.77 ^bc^	1.82 ^c^	12.78 ^a^	4.53 ^bc^	0.25 ^b–d^	287.50 ^fg^
8	0	2.45 ^f–h^	0.64 ^i–k^	2.54 ^jk^	2.35 ^j–l^	0.11 ^j–l^	399.33 ^bc^
	30	2.45 ^f–h^	0.74 ^h–j^	7.45 ^fg^	3.01 ^hi^	0.17 ^f–i^	372.00 ^d^
	60	2.47 ^fg^	1.15 ^d–g^	8.17 ^ef^	4.03 ^c–e^	0.20 ^d–h^	342.50 ^e^
	90	2.46 ^f–h^	1.20 ^de^	12.32 ^ab^	4.19 ^c–e^	0.22 ^c–f^	310.50 ^fg^
12	0	2.14 ^jk^	0.50 ^jk^	1.85 ^kl^	2.25 ^kl^	0.09 ^kl^	411.33 ^b^
	30	2.23 ^ij^	0.59 ^jk^	6.76 ^gh^	2.91 ^h–j^	0.15 ^h–j^	384.00 ^cd^
	60	2.35 ^g–i^	1.00 ^e-h^	7.48 ^fg^	3.93 ^d–f^	0.18 ^e–i^	354.50 ^e^
	90	2.32 ^hi^	1.17 ^d–f^	11.63 ^bc^	4.09 ^c–e^	0.20 ^d–h^	322.50 ^f^
16	0	1.73 ^n^	0.42 ^k^	1.31 ^l^	2.05 ^l^	0.07 ^l^	434.33 ^a^
	30	1.86 ^mn^	0.52 ^jk^	6.22 ^h^	2.71 ^i–k^	0.13 ^i–k^	407.00 ^b^
	60	1.97 ^lm^	0.95 ^e–h^	6.94 ^gh^	3.73 ^e–g^	0.16 ^g–j^	377.50 ^d^
	90	2.08 ^kl^	0.88 ^g–i^	11.09 ^c^	3.89 ^d–g^	0.18 ^e–i^	345.50 ^e^
Significance							
Salinity		**	**	**	**	**	**
MEL		**	**	**	**	**	**
Salinity × MEL		*	**	*	*	**	*

MEL: melatonin, Chl: chlorophyll, P_n_: net photosynthesis rate, Tr: transpiration rate, g_s_: stomatal conductance, and C_i_: sub-stomatal CO_2_ concentration. Values in the same column followed by different letters indicate statistical significance at *p* ≤ 0.05 according to LSD test. * and **: significant at *p* ≤ 0.05 and *p* ≤ 0.01, respectively. Data shown are means of three replicates per treatment.

**Table 2:** Effects of MEL foliar applications (0, 30, 60 and 90 µM) on the shoot and root K and Na concentrations, translocation and selective transport in camelina grown under different levels of salinity (0.5, 4, 8, 12 and 16 dS m^−1^).

Salinity Level (dS m^−1^)	MEL Concentrations (µM)	K (mg g^−1^ DW)		Na (mg g^−1^ DW)		K/Na		Translocation		Selective Transport
		Shoot	Root	Shoot	Root	Shoot	Root	K (mg g^−1^ DW)	Na (mg g^−1^ DW)	
0.5	0	8.78 ^c–e^	2.89 ^b^	2.00 ^f^	3.46 ^f^	4.39 ^d^	0.83 ^c^	3.29 ^e^	0.57 ^g^	4.94 ^fg^
	30	9.37 ^bc^	2.91 ^b^	1.20 ^fg^	2.65 ^fg^	7.88 ^c^	1.10 ^b^	3.89 ^de^	0.45 ^h^	4.14 ^g^
	60	9.69 ^b^	2.49 ^d^	0.71 ^g^	2.17 ^g^	13.71 ^b^	1.14 ^b^	3.21 ^ef^	0.32 ^i^	4.54 ^fg^
	90	11.36 ^a^	3.44 ^a^	0.48 ^g^	1.94 ^g^	23.57 ^a^	1.77 ^a^	3.03 ^ef^	0.24 ^j^	5.62 ^fg^
4	0	7.97 ^d–f^	1.73 ^g^	6.53 ^c^	7.99 ^c^	1.22 ^e–h^	0.21 ^g^	3.38 ^e^	0.81 ^b–d^	4.80 ^fg^
	30	7.92 ^e–f^	2.37 ^e^	6.03 ^cd^	7.49 ^cd^	1.31 ^e–h^	0.31 ^f^	3.58 ^de^	0.80 ^cd^	5.82 ^ef^
	60	8.83 ^b–d^	2.46 ^d^	3.83 ^e^	5.29 ^e^	2.31 ^ef^	0.46 ^e^	3.33 ^e^	0.72 ^e^	5.24 ^fg^
	90	9.05 ^bc^	2.67 ^c^	3.23 ^e^	4.68 ^e^	2.87 ^de^	0.57 ^d^	4.60 ^d^	0.68 ^f^	7.46 ^d^
8	0	6.03 ^h^	0.74 ^k^	7.87 ^ab^	9.33 ^ab^	0.77 ^f–h^	0.08 ^j^	3.76 ^de^	0.84 ^ab^	4.95 ^fg^
	30	5.55 ^h^	0.87 ^j^	6.68 ^c^	8.14 ^c^	0.83 ^f–h^	0.10 ^ij^	4.04 ^de^	0.82 ^bc^	5.09 ^fg^
	60	7.82 ^fg^	1.93 ^f^	4.15 ^e^	5.61 ^e^	1.88 ^e–h^	0.34 ^f^	6.38 ^c^	0.74 ^e^	7.15 ^de^
	90	8.92 ^bc^	2.37 ^e^	4.13 ^e^	5.59 ^e^	2.17 ^e–g^	0.42 ^e^	8.08 ^b^	0.73 ^e^	7.30 ^de^
12	0	5.60 ^h^	0.60 ^lm^	7.74 ^b^	9.20 ^b^	0.72 ^f–h^	0.06 ^j^	6.66 ^c^	0.84 ^ab^	5.46 ^fg^
	30	7.38 ^fg^	0.75 ^k^	5.95 ^cd^	7.40 ^cd^	1.24 ^e–h^	0.10 ^ij^	6.76 ^c^	0.80 ^cd^	7.77 ^d^
	60	6.95 ^g^	1.95 ^f^	5.50 ^d^	6.83 ^d^	1.32 ^e–h^	0.10 ^ij^	9.76 ^a^	0.78 ^d^	8.35 ^cd^
	90	7.89 ^f^	1.21 ^h^	5.34 ^d^	6.79 ^d^	1.51 ^e–h^	0.17 ^gh^	9.43 ^a^	0.78 ^d^	9.59 ^c^
16	0	3.29 ^i^	0.52 ^m^	8.88 ^a^	10.34 ^a^	0.37 ^h^	0.05 ^j^	4.54 ^d^	0.85 ^a^	11.20 ^b^
	30	4.10 ^i^	0.67 ^kl^	8.02 ^ab^	9.48 ^ab^	0.51 ^gh^	0.07 ^j^	2.12 ^f^	0.84 ^ab^	12.15 ^ab^
	60	4.15 ^i^	1.04 ^i^	7.81 ^b^	9.27 ^b^	0.53 ^gh^	0.21 ^gh^	6.30 ^c^	0.84 ^ab^	11.89 ^ab^
	90	5.50 ^h^	1.18 ^h^	5.73 ^cd^	7.19 ^cd^	0.96 ^f–h^	0.16 ^gh^	6.26 ^c^	0.79 ^ab^	13.26 ^a^
Significance										
Salinity		**	**	**	**	**	**	**	**	**
MEL		**	**	**	**	**	**	**	**	**
Salinity × MEL		*	*	*	**	**	**	**	**	**

MEL: melatonin. Values in the same column followed by different letters indicate statistical significance at *p* ≤ 0.05 according to LSD test. * and **: significant at *p* ≤ 0.05 and *p* ≤ 0.01, respectively. Data shown are means of three replicates per treatment.

**Table 3:** Effects of MEL foliar applications (0, 30, 60, and 90 µM) on the fatty acid composition of camelina seeds in plants grown under different levels of salinity (0.5, 4, 8, 12, and 16 dS m^−1^).

Salinity Level(dS m^−1^)	MEL Concentrations (µM)	SFA		MUFA			PUFA			
		C16: 0	C18: 0	C18: 1	C20: 1	C22: 1	C18: 2	C18: 3	C20: 2	C20: 3
0.5	0	10.37 ^e^	1.44 ^f^	16.34 ^b–d^	2.33 ^e^	0.25 ^d^	23.55 ^e^	47.66 ^b^	0.36 ^g–i^	0.18 ^fg^
	30	10.86 ^e^	0.64 ^g^	16.85 ^bc^	3.85 ^d^	0.38 ^c^	29.00 ^d^	47.66 ^b^	1.49 ^e^	0.82 ^c^
	60	10.87 ^e^	0.52 ^g^	17.39 ^b^	5.02 ^c^	1.42 ^b^	30.29 ^c^	49.30 ^a^	3.77 ^c^	1.30 ^b^
	90	10.64 ^e^	1.36 ^f^	21.13 ^a^	7.00 ^a^	1.61 ^a^	37.74 ^a^	49.46 ^b^	4.00 ^b^	1.81 ^a^
4	0	13.24 ^d^	1.77 ^e^	13.75 ^f^	0.55 ^g^	0.00 ^e^	22.30 ^f^	34.13 ^g^	0.23 ^hi^	0.16 ^g^
	30	14.73 ^c^	1.39 ^f^	15.32 ^de^	1.23 ^f^	0.00 ^e^	28.33 ^d^	35.42 ^f^	0.41 ^gh^	0.37 ^de^
	60	12.48 ^d^	1.40 ^f^	16.08 ^cd^	3.85 ^d^	0.38 ^c^	30.24 ^c^	41.14 ^cd^	2.63 ^d^	0.40 ^d^
	90	10.26 ^e^	1.95 ^de^	17.06 ^bc^	5.86 ^b^	0.47 ^c^	31.66 ^b^	48.82 ^a^	5.32 ^a^	1.33 ^b^
8	0	15.69 ^c^	2.49 ^bc^	3.90 ^g^	0.52 ^gh^	0.00 ^e^	21.59 ^f^	15.61 ^h^	0.23 ^hi^	0.05 ^h^
	30	23.38 ^a^	2.08 ^d^	13.90 ^f^	0.63 ^g^	0.00 ^e^	22.14 ^f^	37.28 ^e^	0.20 ^i^	0.22 ^f^
	60	18.89 ^b^	2.37 ^c^	14.56 ^ef^	2.86 ^e^	0.00 ^e^	23.72 ^e^	40.69 ^d^	0.42 ^gh^	0.33 ^e^
	90	13.42 ^d^	2.61 ^b^	14.56 ^ef^	4.77 ^c^	0.20 ^d^	23.72 ^e^	42.17 ^c^	0.82 ^f^	0.42 ^d^
12	0	-	-	-	-	-	-	-	-	-
	30	-	-	-	-	-	-	-	-	-
	60	-	-	-	-	-	-	-	-	-
	90	1.72 ^f^	26.68 ^a^	4.43 ^g^	2.84 ^e^	0.00 ^e^	20.11 ^g^	41.26 ^cd^	0.52 ^g^	0.33 ^e^
Significance										
Salinity		**	**	**	**	**	**	**	**	**
MEL		**	**	**	**	**	**	**	**	**
Salinity × MEL		**	**	**	**	**	**	**	**	**

Values in the same column followed by different letters indicate statistical significance at *p* ≤ 0.05 according to LSD test. **: significant at *p* ≤ 0.01. Data shown are means of three replicates per treatment.

## Data Availability

Not applicable.
